# Author Correction: The role and risks of selective adaptation in extreme coral habitats

**DOI:** 10.1038/s41467-024-44817-y

**Published:** 2024-01-17

**Authors:** Federica Scucchia, Paul Zaslansky, Chloë Boote, Annabelle Doheny, Tali Mass, Emma F. Camp

**Affiliations:** 1 Department of Marine Biology, Leon H, Charney school of Marine Sciences, University of Haifa, Haifa, Israel; 2 Department for Operative, Preventive and Pediatric Dentistry, Charité-Universitätsmedizin, Berlin, Germany; 3 Climate Change Cluster, University of Technology Sydney, Ultimo, NSW Australia

**Keywords:** Biodiversity, Conservation biology, Marine biology

Correction to: *Nature Communications* 10.1038/s41467-023-39651-7, published online 28 July 2023

The original version of this Article contained an error in the calculation of the density of reef corals, leading to errors in the density values shown in Fig. 3e and the Source Data for Fig. 3e, and in statistical results reported in the legend for Fig. 3 and in the Results section. The correct version of Fig. 3 is:


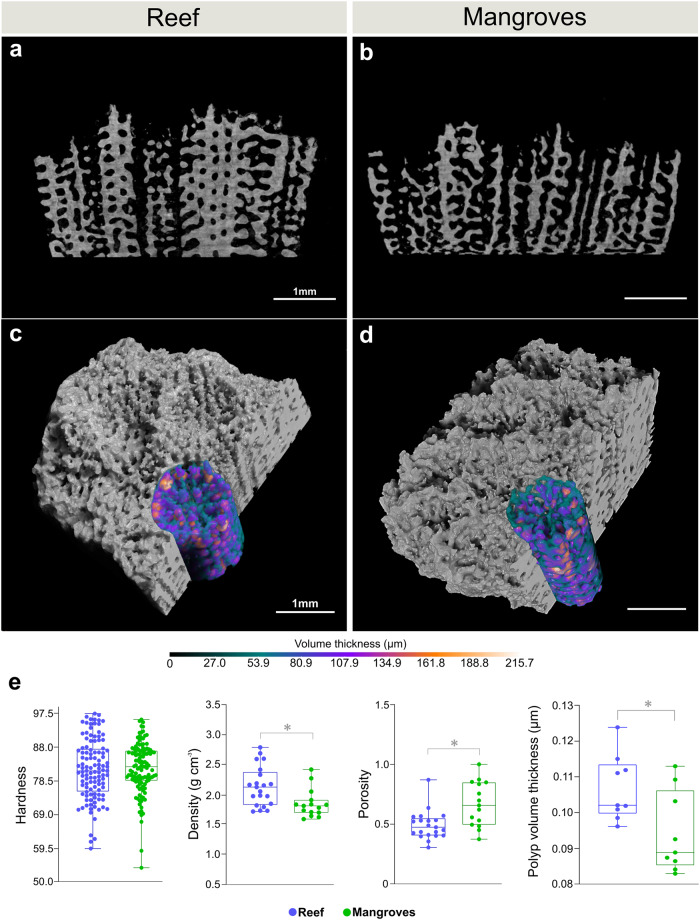


which replaces the previous incorrect version:


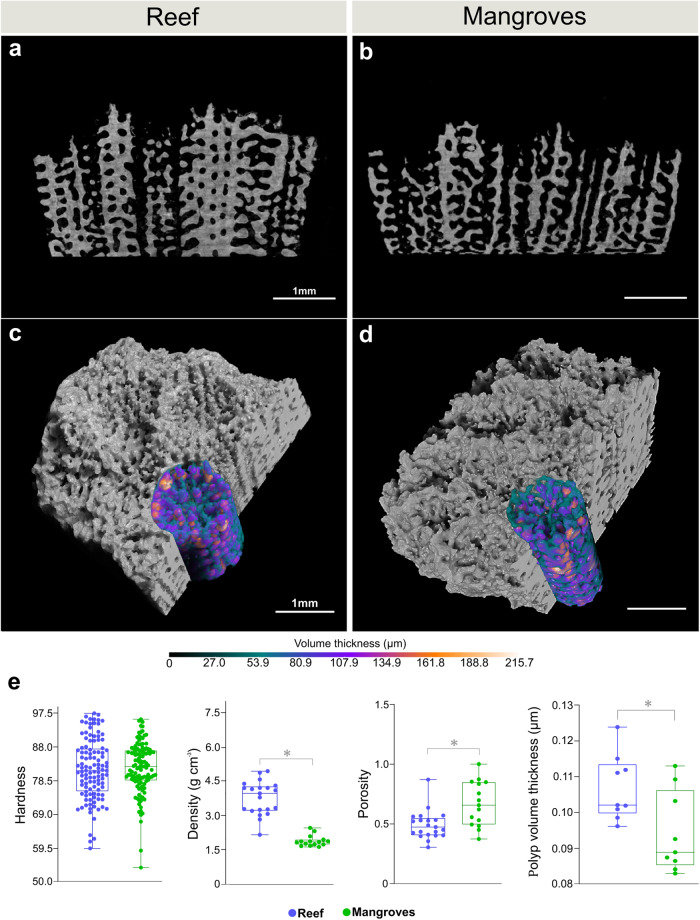


The legend of Fig. 3 for panel (e) incorrectly read “Mann–Whitney test (density, *n* = 15–21, *p* < 0.0001”. The correct version states ‘*p* < 0.01’ in place of ‘*p* < 0.0001’. The Results subsection ‘Morphological adjustments to an extreme environment’ incorrectly read “lower density (Mann–Whitney test, *p* < 0.0001) compared to reef corals”. The correct version states ‘*p* < 0.01’ in place of ‘*p* < 0.0001’.These errors have now been corrected in both the HTML and the PDF versions of the Article. The HTML has been updated to include a corrected version of the Source Data; the original incorrect version of the Source Data can be found as Supplementary Information associated with this Correction.

### Supplementary information

Updated Source Data

Incorrect Source Data

