# Supplementary material for: The role and risks of selective adaptation in extreme coral habitats
Source: Nat Commun. 2023 Jul 28;14:4475. doi: 10.1038/s41467-023-39651-7 (PMC10382478; doi:10.1038/s41467-023-39651-7)
Supplement: Supplementary file 1 — Supplementary Information [file 41467_2023_39651_MOESM1_ESM.docx]

**The role and risks of selective adaptation in extreme coral habitats**

Federica Scucchia^1*^, Paul Zaslansky^2^, Chloe Boote^3^, Annabelle Doheny^3^, Tali Mass^1&^

and Emma F. Camp^3*&^

^1^ Department of Marine Biology, Leon H, Charney school of Marine Sciences University of Haifa, Israel

^2^ Department for Operative and Preventive Dentistry, Charité-Universitätsmedizin, Berlin, Germany

^3^ Climate Change Cluster, University of Technology Sydney, Ultimo, NSW, Australia

*Corresponding authors: Federica Scucchia, [fscucchia@ufl.edu](mailto:fscucchia@ufl.edu); Emma F. Camp, [emma.camp@uts.edu.au](mailto:emma.camp@uts.edu.au)

^&^These authors contributed equally

**Supplementary Information**

Figure 1. Overview of the RNA-Seq differential expression results.

Figure 2. Hardness measurements from internal and external regions of skeleton cross-sections.

Figure 3. Differential expression of biomineralization-related genes across habitats.

Figure 4. Overview of single-polyp volumetric thickness analysis.

Table 1. Abiotic conditions of mangrove and reef sites in the study area and in other locations reported in the literature.

Table 2. Proteomes databases employed to detect the species origin of coral and algal symbionts sequences.

Movie 1. Tomographic cross-sectional slices and three-dimensional rendering of the skeleton volumetric thickness in an example skeleton fragment.

Supporting References


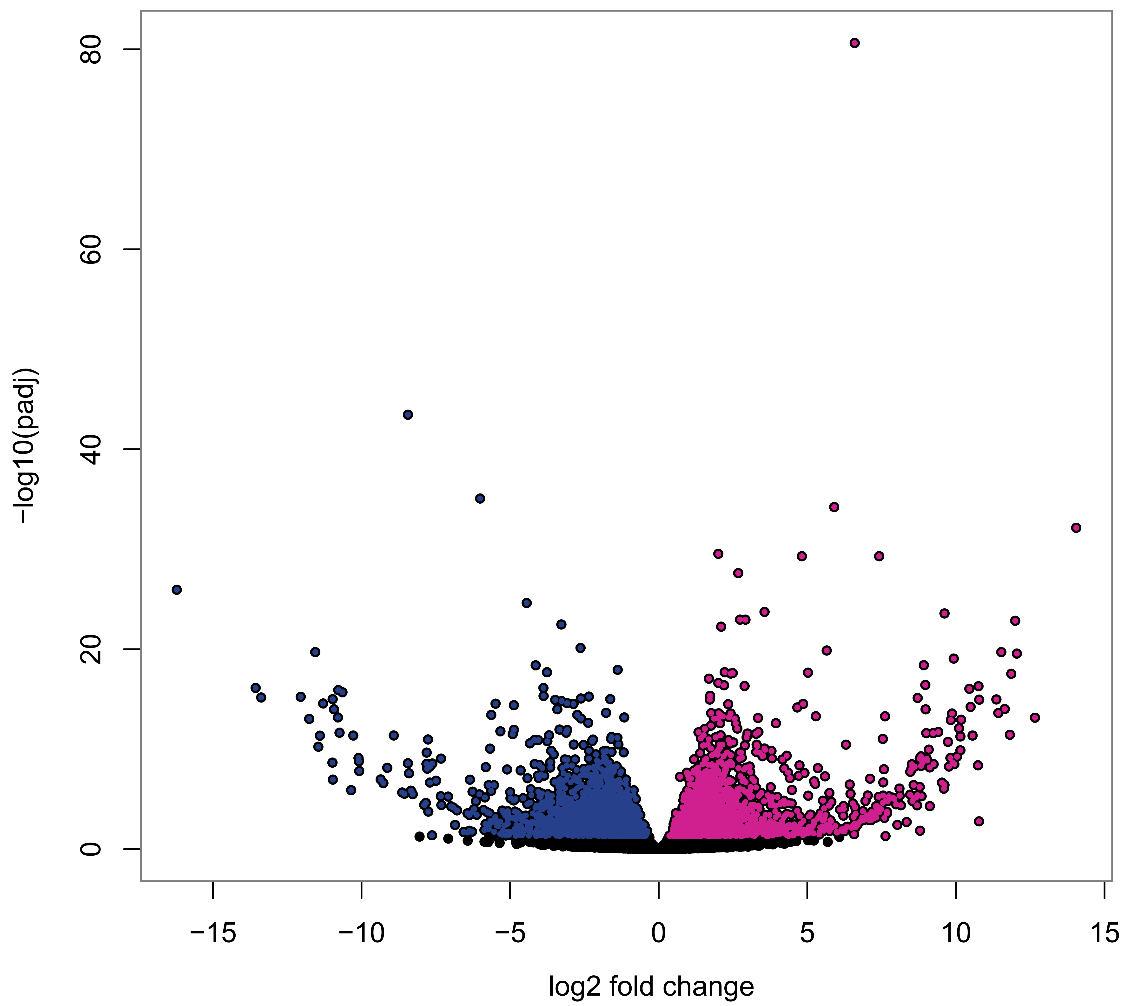


**Supplemental Figure 1. Overview of the RNA-Seq differential expression results.** Volcano plot showing all expresses genes found in mangrove corals as compared to reef corals, with blue and pink indicating differentially expressed genes that are significantly (Wald test, p < 0.05) down-regulated or up-regulated, respectively.


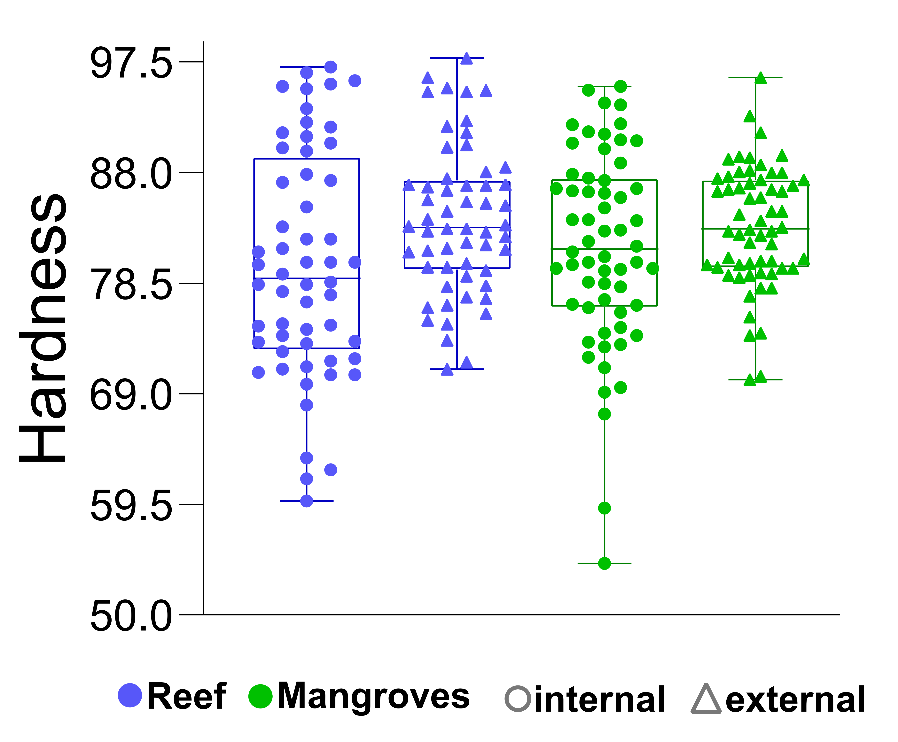


**Supplemental Figure 2. Hardness measurements from internal and external regions of skeleton cross-sections.** A ~1 cm cross section of coral was taken from the center of each sample (n = 3 samples per study site). Micro-hardness was assessed per each cross section with 40 replicate measurements per sample; 20 from the external 5 mm and 20 from the internal 5 mm to account for differences in old versus new growth. Hardness values are measured on a calibrated scale which gives the Shore hardness value (SH) in its own units, with higher values indicating a harder material. Boxplots show first and third quartiles, median line and whiskers at ±1.5 interquartile range. No statistical difference was found in old versus new growth between reef and mangrove corals (two-sided unpaired t-test, p > 0.05). Source data are provided as a Source Data file.


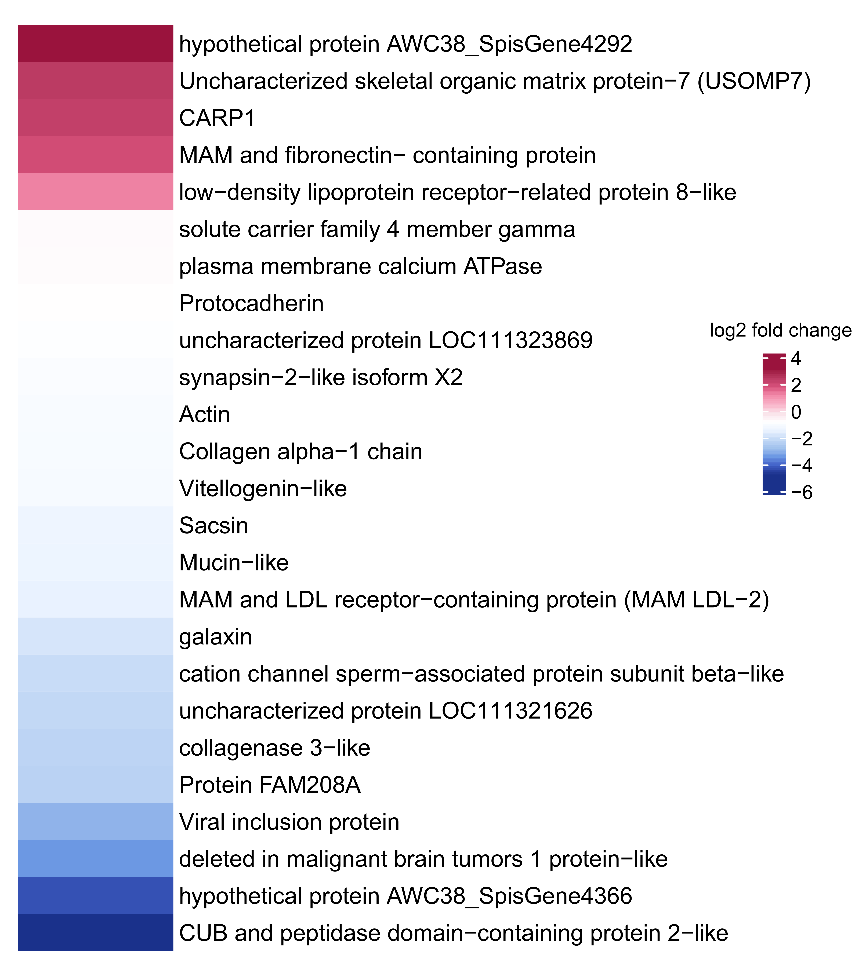


**Supplemental Figure 3. Differential expression of biomineralization-related genes across habitats.** Heatmap showing the log2 fold change of significantly (Wald test, p value < 0.05) up-regulated and down-regulated biomineralization-related genes detected in mangroves as compared to reef *P. lutea* corals. See Supplemental Data 2 for the full list of genes.


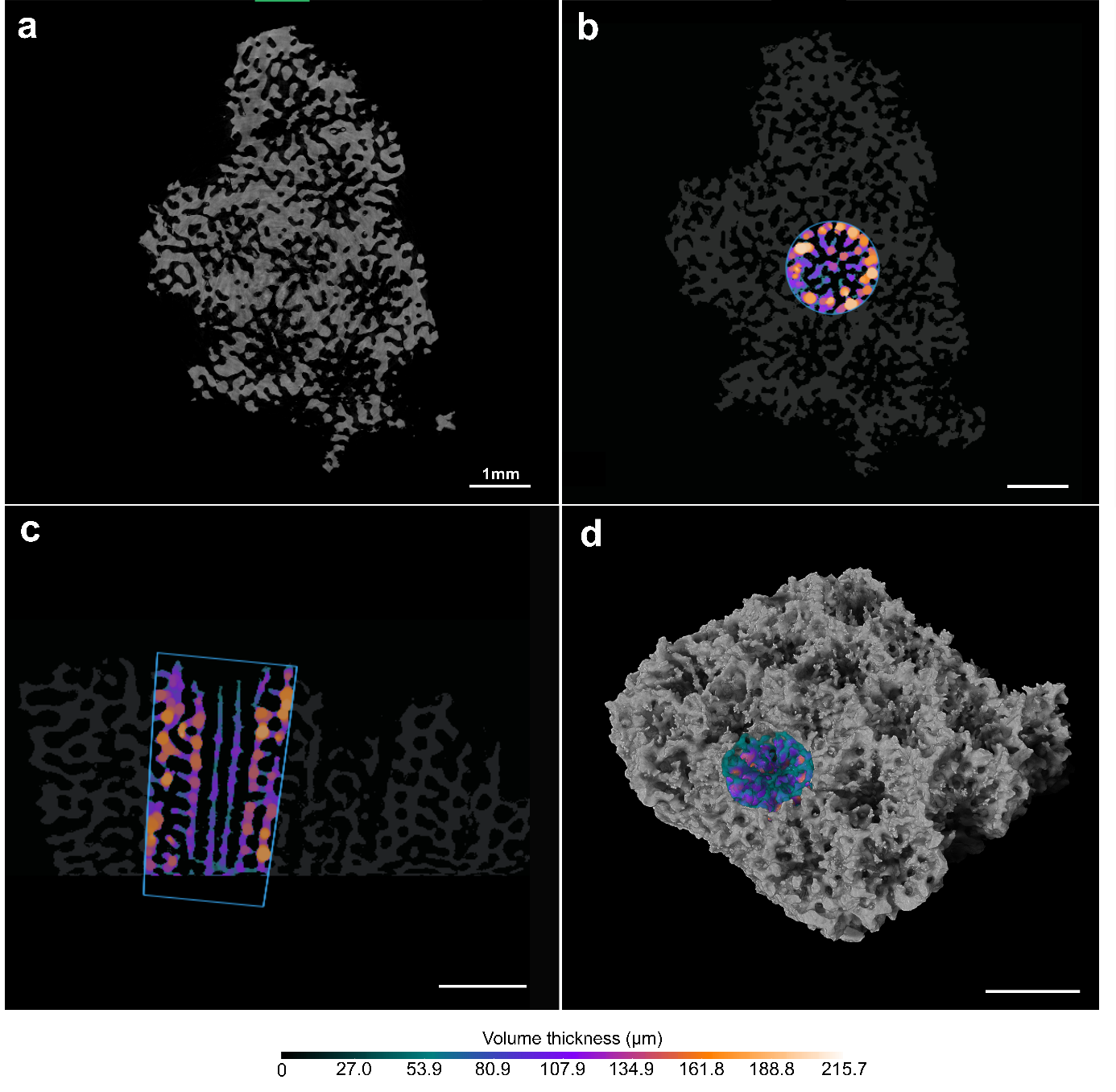


**Supplemental Figure 4. Overview of single-polyp volumetric thickness analysis. a** Cross-sectional tomography slice (transverse plane) showing the internal skeletal architecture in an example *P. lutea* coral fragment. **b**, **c** Volumetric thickness distribution of a single polyp in the (**b**) transverse and (**c**) longitudinal planes. The linearity of the polyps’ walls across the entire skeleton of *P. lutea* allows for clear distinction and selection of each single polyp. **d** 3D rendering of the skeleton showing the top-view of the single-polyp thickness distribution. Thickness values are 2 dimensional measurements of the thickness of each voxel (volume pixel) in the region being measured. All tomographic data was acquired with a resolution of 3.61 µm. Scale bars: 1 mm.

**Supplemental Table 1. Abiotic conditions of mangrove and reef sites in the study area and in other locations reported in the literature.** Measurements of abiotic factors carried out in the study sites of this work (Woody Isles and Low Isles, in italics) and in other locations across the world. Measurements in the table are reported only for studies directly comparing mangrove and reef sites. Common dynamics in abiotic conditions observed in mangrove environments include higher temperatures, higher or lower salinity, lower or similar light levels, and lower oxygen and pH levels compared to neighboring reef sites^1–6^.


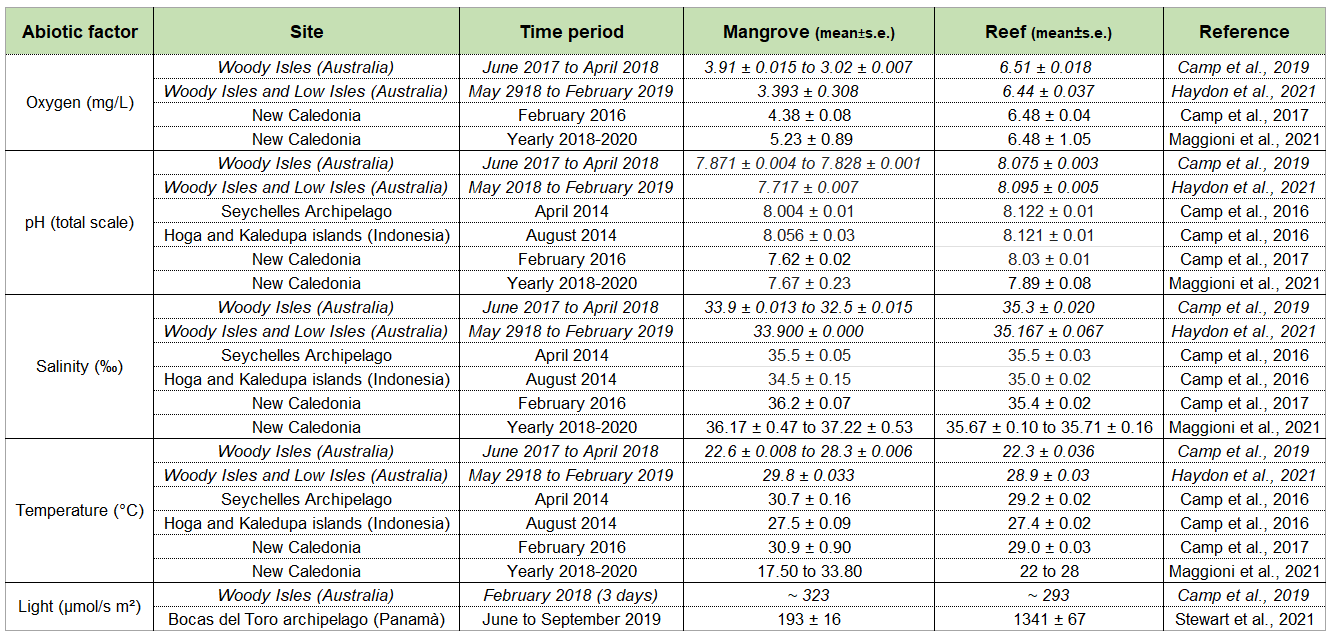


**Supplemental Table 2. Proteomes databases employed to detect the species origin of coral and algal symbionts sequences.**


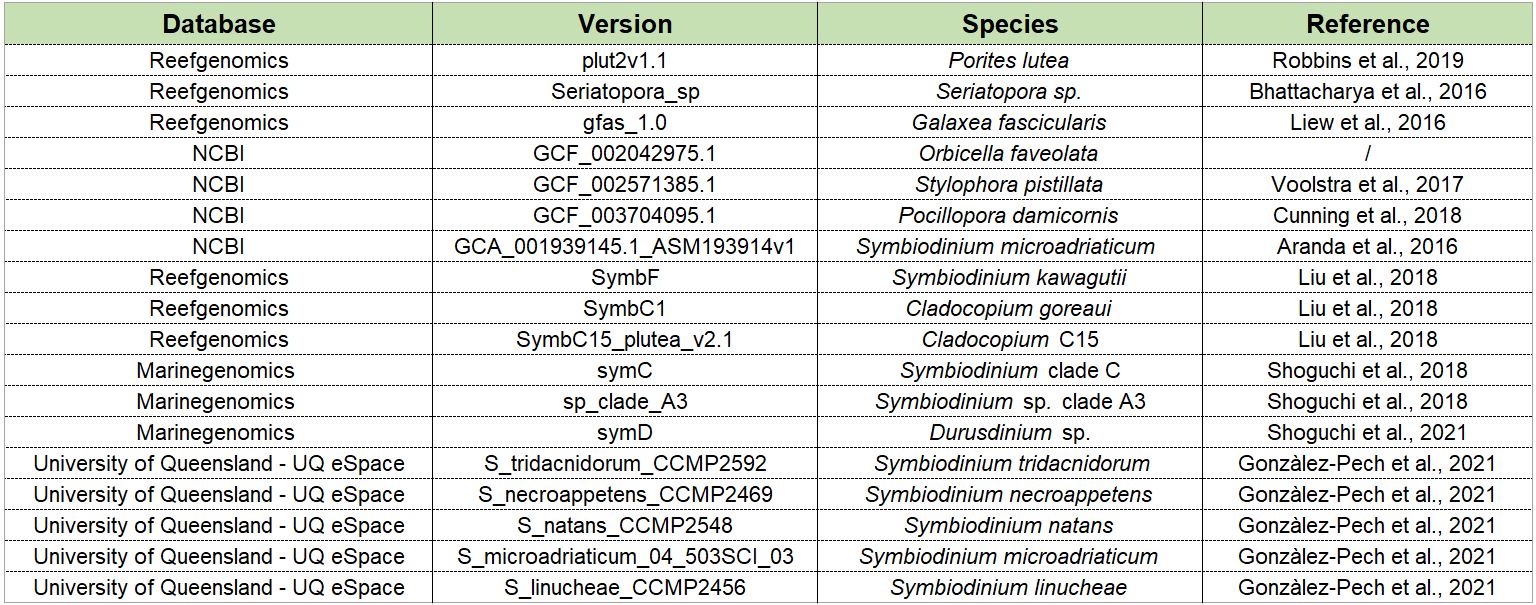


**References**

1. Camp, E. *et al.* Mangrove lagoons of the Great Barrier Reef support coral populations persisting under extreme environmental conditions. *Mar. Ecol. Prog. Ser.* **625**, 1–14 (2019).

2. Camp, E. F. *et al.* Reef-building corals thrive within hot-acidified and deoxygenated waters. *Sci Rep* **7**, 2434 (2017).

3. Camp, E. F. *et al.* Mangrove and Seagrass Beds Provide Different Biogeochemical Services for Corals Threatened by Climate Change. *Front. Mar. Sci.* **3**, (2016).

4. Stewart, H. A., Kline, D. I., Chapman, L. J. & Altieri, A. H. Caribbean mangrove forests act as coral refugia by reducing light stress and increasing coral richness. *Ecosphere* **12**, (2021).

5. Maggioni, F. *et al.* The Bouraké semi-enclosed lagoon (New Caledonia) – a natural laboratory to study the lifelong adaptation of a coral reef ecosystem to extreme environmental conditions. *Biogeosciences* **18**, 5117–5140 (2021).

6. Stewart, H. A. *et al.* Novel coexisting mangrove-coral habitats: Extensive coral communities located deep within mangrove canopies of Panama, a global classification system and predicted distributions. *PLoS ONE* **17**, e0269181 (2022).
