# Supplementary material for: The role and risks of selective adaptation in extreme coral habitats
Source: Nat Commun. 2023 Jul 28;14:4475. doi: 10.1038/s41467-023-39651-7 (PMC10382478; doi:10.1038/s41467-023-39651-7)
Supplement: Supplementary file 3 — Description of Additional Supplementary Files [file 41467_2023_39651_MOESM3_ESM.pdf]

Description of Additional Supplementary Files

Supplementary Data 1: Full list of enriched GO terms and associated slim categories.

Supplementary Data 2: Known coral biomineralization-related proteins. Accession numbers/gene IDs of know biomineralization-related proteins from new coral skeletal proteome data[8] and from the literature[9–24]. The first reference in the 'References' column is the one related to the accession number/gene ID, all other references relate to studies where that specific gene/protein was previously detected/immunolocalized (chronological order). These known coral biomineralization-related proteins have been previously listed in[25]. See Supplementary Information for the list of references.

Supplementary Movie 1: Tomographic cross-sectional slices and three-dimensional rendering of the skeleton volumetric thickness in an example skeleton fragment.
