# Supplementary material for: The role and risks of selective adaptation in extreme coral habitats
Source: Nat Commun. 2023 Jul 28;14:4475. doi: 10.1038/s41467-023-39651-7 (PMC10382478; doi:10.1038/s41467-023-39651-7)
Supplement: Supplementary file 7 — Reporting Summary [file 41467_2023_39651_MOESM7_ESM.pdf]

nature portfolio

Corresponding author(s): Federica Scucchia, Emma F. Camp

Last updated by author(s): Jun 1, 2023

Reporting Summary

Nature Portfolio wishes to improve the reproducibility of the work that we publish. This form provides structure for consistency and transparency in reporting. For further information on Nature Portfolio policies, see our Editorial Policies and the Editorial Policy Checklist.

Statistics

For all statistical analyses, confirm that the following items are present in the figure legend, table legend, main text, or Methods section.

n/a	Confirmed	
☐	☒	The exact sample size (*n*) for each experimental group/condition, given as a discrete number and unit of measurement
☐	☒	A statement on whether measurements were taken from distinct samples or whether the same sample was measured repeatedly
☐	☒	The statistical test(s) used AND whether they are one- or two-sided<br>*Only common tests should be described solely by name; describe more complex techniques in the Methods section.*
☐	☒	A description of all covariates tested
☐	☒	A description of any assumptions or corrections, such as tests of normality and adjustment for multiple comparisons
☐	☒	A full description of the statistical parameters including central tendency (e.g. means) or other basic estimates (e.g. regression coefficient) AND variation (e.g. standard deviation) or associated estimates of uncertainty (e.g. confidence intervals)
☐	☒	For null hypothesis testing, the test statistic (e.g. *F*, *t*, *r*) with confidence intervals, effect sizes, degrees of freedom and *P* value noted<br>*Give P values as exact values whenever suitable.*
☒	☐	For Bayesian analysis, information on the choice of priors and Markov chain Monte Carlo settings
☒	☐	For hierarchical and complex designs, identification of the appropriate level for tests and full reporting of outcomes
☒	☐	Estimates of effect sizes (e.g. Cohen's *d*, Pearson's *r*), indicating how they were calculated

Our web collection on statistics for biologists contains articles on many of the points above.

Software and code

Policy information about availability of computer code

Data collection	All programs were used for data analysis, not collection.
Data analysis	Cutadapt (v2.6), Trimmomatic (v0.39) and SortMeRNA (v4.3.6), Diamond (v2.0.11), HISAT2 (v2.2.1), StringTie (v2.2.5), GFFcompare (v0.12.2), R (v3.6.3), DESeq2 (v1.26.0), R package NbClust (v3.0), R package Goseq (v1.42.0), R package GSEABase (v1.52.1), R package GSEABase (v1.52.1), GATK (v4.2.0), PLINK (v2.0), R package SNPRelate (v1.20.1), R package HIERFSTAT (v0.5.10), nRecon (v 1.7.4.2, Brucker micro-CT), Dragonfly (v2021.3), GraphPad Prism (v9.0.0).<br>All the scripts employed to analyze the RNA-seq data are accessible through the electronic notebook https://github.com/fscucchia/Plutea_mangrove_reef.

For manuscripts utilizing custom algorithms or software that are central to the research but not yet described in published literature, software must be made available to editors and reviewers. We strongly encourage code deposition in a community repository (e.g. GitHub). See the Nature Portfolio guidelines for submitting code & software for further information.

Data

Policy information about availability of data

All manuscripts must include a data availability statement. This statement should provide the following information, where applicable:

- Accession codes, unique identifiers, or web links for publicly available datasets
- A description of any restrictions on data availability
- For clinical datasets or third party data, please ensure that the statement adheres to our policy

The morphological data generated in this study have been deposited in the Zenodo database [https://doi.org/10.5281/zenodo.7454382]. Transcriptomic data generated in this study have been deposited in the National Center for Biotechnology Information under accession code PRJNA912580 [https://www.ncbi.nlm.nih.gov/bioproject/?term=PRJNA912580]. Morphological and genetic data generated are also provided in the Supplementary Information/Source Data file. All the scripts employed to analyze the RNA-seq data are accessible through the Github electronic notebook [https://github.com/fscucchia/Plutea_mangrove_reef].

Human research participants

Policy information about studies involving human research participants and Sex and Gender in Research.

Reporting on sex and gender	not applicable
Population characteristics	not applicable
Recruitment	not applicable
Ethics oversight	not applicable

Note that full information on the approval of the study protocol must also be provided in the manuscript.

Field-specific reporting

Please select the one below that is the best fit for your research. If you are not sure, read the appropriate sections before making your selection.

☐ Life sciences ☐ Behavioural & social sciences ☒ Ecological, evolutionary & environmental sciences

For a reference copy of the document with all sections, see nature.com/documents/nr-reporting-summary-flat.pdf

Ecological, evolutionary & environmental sciences study design

All studies must disclose on these points even when the disclosure is negative.

Study description	Porites lutea corals at two different sites (Woody Isles and Low Isles, Australia) were sampled at a depth of 1-1.5 m. Colonies (9-10 per site) of comparable size were sampled with a chisel (5-6 m apart to minimize the potential of sampling clonal genotypes) to obtain small colonies (4-6 cm total length) at both sites. Collected corals were assigned to molecular analysis or skeleton physical property analysis. Four independent samples were obtained for each site for molecular analysis. Multiple tests were performed to assess the physical properties of the coral skeletons. Bulk density and porosity were determined for 5-6 samples per site (3 technical replicates per sample). For hardness, a ~1 cm cross section of coral was taken from the center of each sample (same location across samples, n = 3 samples per study site). Tomographic scanning of the skeleton fragments was conducted on n = 3 per site.
Research sample	The genus Porites is considered a hardy coral taxon and has been documented across extreme mangrove systems globally, making it a good candidate to test the resilience of corals to extreme environmental changes. Porites lutea is an important reef forming species in the Indo-Pacific, and it is a dominant coral at the Woody Isles mangrove lagoon and at the Low Isle reef on the Great Barrier Reef. For this study, we collected from each study site 8 colonies of P. lutea , which we analyzed as representative of the local population living at both Woody and Low Isles.
Sampling strategy	Corals at the reef and mangrove sites were sampled at a depth of 1-1.5 m. Colonies (9-10 per site) of comparable size were sampled with a chisel (5-6 m apart to minimize the potential of sampling clonal genotypes) to obtain small colonies (4-6 cm total length) at both sites. Sample size was determined in order to have enough replicates per each site (> 3) for all subsequent analyses to be performed (molecular and skeletal). Sensitivity of the location and permitting restrictions limited overcollection, which also shaped the sample size used.
Data collection	Corals were collected in February 2018 by Emma Camp.
Timing and spatial scale	Samples were collected on the 21st February 2018. Independent colonies (> 5 m apart to minimize the potential of sampling clonal genotypes) were collected at each of the two sites (Low Isles reef and Woody Isles mangrove lagoon).
Data exclusions	No data were excluded

Reproducibility	Experimental design and analyses performed are described in details to facilitate reproducibility of the experimental findings. In addition, all code used and raw data are provided along with the manuscript.
Randomization	Independent colonies (9-10 per site, > 5 m apart to minimize the potential of sampling clonal genotypes) were haphazardly collected at each site. Per each habitat of origin, colonies were randomly assigned to either molecular analysis (4 per site) or skeleton physical property analysis (5-6 per site).
Blinding	Coral samples were collected haphazardly within each environment. Blinding was not relevant to this study, since divers had to know in which environment they were going to dive to collect the samples.

Did the study involve field work? ☒ Yes ☐ No

Field work, collection and transport

Field conditions	Samples were collected during the summer, wet season. Conditions were dry when the corals were collected. Average temperature at Low Isles reef at time of collection. Longterm site data for temperature, pH, oxygen and salinity is published in Camp et al., 2019; Mangrove lagoons of the Great Barrier Reef support coral populations persisting under extreme environmental conditions.
Location	Woody Isles and Low Isles Reef (16.388°S, 145.566°E). The Woody Isles site is a semi-enclosed lagoon surrounded by mangrove forest that undergoes daily tidal flushing. The shallow (0-2.5 m) nature of the lagoon in combination with rich nutrient cycling and associated microbial activity results in highly variable abiotic conditions that are characteristic of mangrove systems. Samples were collected in both sites from a depth of 1-1.5 m.
Access & import/export	The site was accessed by boat to limit any impact to the reef. All coral collections were undertaken in accordance with the Great Barrier Reef Marine Park Authority rules. The collection permit was G18/40023.1 issued to Emma Camp. In accordance with the permit, a limit number of samples, and size of sample were collected to minimize impact on the site. Community consultation, including with traditional owners occurred before th permit was issued.
Disturbance	As described above, we followed all permitting requirements to minimise any impact on the site. Further, coral collections were done by hand to avoid any potential damage from power tools.

Reporting for specific materials, systems and methods

We require information from authors about some types of materials, experimental systems and methods used in many studies. Here, indicate whether each material, system or method listed is relevant to your study. If you are not sure if a list item applies to your research, read the appropriate section before selecting a response.

Materials & experimental systems

n/a	Involved in the study	
☒	☐	Antibodies
☒	☐	Eukaryotic cell lines
☒	☐	Palaeontology and archaeology
☐	☒	Animals and other organisms
☒	☐	Clinical data
☒	☐	Dual use research of concern

Methods

n/a	Involved in the study	
☒	☐	ChIP-seq
☒	☐	Flow cytometry
☒	☐	MRI-based neuroimaging

Animals and other research organisms

Policy information about studies involving animals; ARRIVE guidelines recommended for reporting animal research, and Sex and Gender in Research

Laboratory animals	No laboratory animals
Wild animals	Porites lutea corals at both Woody Isles and Low Isles (Australia) were sampled at a depth of 1-1.5 m. Colonies (9-10 per site) of comparable size were sampled with a chisel (5-6 m apart to minimize the potential of sampling clonal genotypes) to obtain small colonies (4-6 cm total length) at both sites. Samples were placed in a zip-lock bag containing native seawater to return to the research vessel (< 20 min). Once on the research vessel, colonies were assigned to molecular analysis or skeleton physical property analysis.
Reporting on sex	Sex information is not relevant here, thus it has not been collected.
Field-collected samples	Field-collected corals were immediately frozen in liquid nitrogen and maintained at -80°C at the University of Technology Sydney prior to RNA extraction. Samples used for physical property analysis were air dried and stored in protective containers to prevent

corallite damage prior to physical property analysis.

Ethics oversight	No ethical approval is required for invertebrate organisms.

Note that full information on the approval of the study protocol must also be provided in the manuscript.
